# Detection of a sudden change of the field time series based on the Lorenz system

**DOI:** 10.1371/journal.pone.0170720

**Published:** 2017-01-31

**Authors:** ChaoJiu Da, Fang Li, BingLu Shen, PengCheng Yan, Jian Song, DeShan Ma

**Affiliations:** 1 School of Mathematics and Computer Science Institute, Northwest University for Nationalities, Lanzhou, GanSu, China; 2 College of Atmospheric Sciences, Lanzhou University, Lanzhou, GanSu, China; 3 College of Atmospheric Sciences, Chengdu University of Information Technology, Chengdu, SiChuan, China; 4 Institute of Arid Meteorology of CMA, Lanzhou, GanSu, China; 5 College of Science, Inner Mongolia University of Technology, Hohhot, Inner Mongolia, China; Shanxi University, CHINA

## Abstract

We conducted an exploratory study of the detection of a sudden change of the field time series based on the numerical solution of the Lorenz system. First, the time when the Lorenz path jumped between the regions on the left and right of the equilibrium point of the Lorenz system was quantitatively marked and the sudden change time of the Lorenz system was obtained. Second, the numerical solution of the Lorenz system was regarded as a vector; thus, this solution could be considered as a vector time series. We transformed the vector time series into a time series using the vector inner product, considering the geometric and topological features of the Lorenz system path. Third, the sudden change of the resulting time series was detected using the sliding *t*-test method. Comparing the test results with the quantitatively marked time indicated that the method could detect every sudden change of the Lorenz path, thus the method is effective. Finally, we used the method to detect the sudden change of the pressure field time series and temperature field time series, and obtained good results for both series, which indicates that the method can apply to high-dimension vector time series. Mathematically, there is no essential difference between the field time series and vector time series; thus, we provide a new method for the detection of the sudden change of the field time series.

## 1 Introduction

In 1953, Hadamard solved the Cauchy problem of Laplace's equation by formulating the instability of the solution of the differential equation for the first time, and constructed the counterexample, which shows that the differential equation is sensitive to the initial value[1]. In the middle of the20th century, Thom studied the singularity theory of a mapping on the differentiable manifold and classified the singularities of the function in Euclidian space to obtain a series of conclusions, which include the famous transversality theorem. These conclusions form the mathematical foundation of sudden change theory[2]. Zeeman conducted a systematic study of sudden change theory and expanded the theory's application[3]. In 1963, Lorenz analyzed the nonlinear effect in the convection motion equation of the atmosphere and found that when the parameters of the equations are particular values, the path motion becomes complex and uncertain, which leads to the unpredictability of the path[4]. The study's significance has two aspects: First, the study confirmed the counterexample of Hadamard from the viewpoint of a numerical experiment. Second, tests conducted during the study illustrated that the climate demonstrates the phenomenon of sudden change.

Generally, a nonlinear dynamical system has two or more equilibrium states. This is true for the dynamical system that derives from the numerical discretization of the atmospheric dynamic equations[5]. Sudden change is the process of a path transforming from one equilibrium state to another[6]. In 1958, Ye, et al. found that atmospheric circulation experienced a sudden change in June and October[7]. Whether considering the atmospheric dynamic model or the real state of the atmosphere, the atmospheric system is chaotic, sensitive to the initial state, and experiences sudden changes. Fu, et al. provided a universal definition of a sudden change of climate: “the sudden change is the jumpy transformation phenomenon from one steady state (the stable and sustainable change trend) to another steady state (the stable and sustainable change trend), the performance is a sharp change from one statistical feature to another statistical feature in space and time”[8], Lian, et al. conducted research on the probable mechanism of low summer temperature and early summer anomalous cold vortex activity in northeast China, and provided the significance precursor signal[9–11]. Feng, et al. detected the sudden change of precipitation and temperature based on dynamic structure sudden change theory[12–15]. Da, et al. conducted research from the viewpoint of numerical weather transitional prediction and indicated the predictability of a sudden turn from drought to flood[16]. This sudden turn is caused by the chaotic character of the atmosphere. The numerical model is sensitive to the initial value and parameters, which leads to difficulties in numerical weather prediction. From information theory, Huang and Feng et al. used historical observation data as the starting point and applied a dynamic statistical method and numerical model to improve the numerical model's results [17–30]. Regarding research on sudden change in the meteorological field, most studies have been based on single time series or have converted the field time series into a single time series using a statistical method, such as the Aleutian low index and Pacific interdecadal oscillation (PDO) index. Few researchers use geometric and topological methods to study the sudden change of the field time series. In this paper, based on the Lorenz system, we propose a new sudden change detection method for the field time series using geometric and topological methods.

## 2 Detection method for a sudden change of the field time series

### 2.1 Field time series

The field time series is

$$X_{1},X_{2},X_{3},\ldots ,X_{n},$$ (1)

where *X*_*i*_ = (*X*_*i*1_, *X*_*i*2_, …, *X*_*im*_) is the moment of the field time series *i*, and *x*_*ij*_ is the value of the moment *i* and the spatial dimension *j*. The meteorological data of the China Meteorological Administration (CMA), with respect to time and space, is a field time series. If *m* = 1, it is a time series:

$$x_{1},x_{2},x_{3},\ldots ,x_{n}.$$ (2)

For Eq (2), the sudden change detection method is mature and credible. In this paper, we provide a new detection method for Eq (1), which is an *n*-dimensional vector. The Lorenz system two features, one, it is a nonlinear differential equation, and the nonlinearity is the source of chaos; second, it has good meteorological background[4], so we take the Lorenz system as an example, the Lorenz system is written as follows:

$$\left\{ \begin{array}{l} \frac{dx}{dt}=10(-x+y)\\ \frac{dy}{dt}=28x-y-xz\\ \frac{dz}{dt}=xy-\frac{8}{3}z. \end{array}\right.$$ (3)

Its numerical solution is a three-dimensional vector time serieswith respect to time. Da conducted a study of the Lorenz system and obtained a boundary surface, which can both differentiate and demarcate the quasi-stable region and quasi-unstable region. In the quasi-stable region, the dynamic characteristics of the Lorenz path are relatively stable, but in the quasi-unstable region, the Lorenz path displays unstable dynamic characteristics. At some times, the Lorenz pathcan run through the boundary surfaceto another equilibrium region[16], which is a sudden change. Given the initial value, a numerical method is applied, the time integral step and integral are fixed, and we calculate the value of every moment of Eq (3) to obtain its numerical solution. Table 1 provides the numerical solution of Eq (3), where the initial field is (12, 23, 56), the integral step is 0.01, and the integral interval is [0,10], which contains the initial field. Consider the numerical solution of the Lorenz system, that is,

$$\alpha_{0}=(x_{0},y_{0},z_{0}),\alpha_{1}=(x_{1},y_{1},z_{1}),\alpha_{2}=(x_{2},y_{2},z_{2}),\alpha_{3}=(x_{3},y_{3},z_{3}),\ldots ,$$ (4)

which is a vector time series. Mathematically, there is no essential difference between the vector time series Eq (4) and the field time series Eq (1). If we can obtain one sudden change detection method for the vector time series and apply it to the field time series, we can obtain a detection method for the sudden change of the field time series.

**Table 1:** Vector time series formed by the numerical solution of the Lorenz system.

t	0	0.01	0.02	0.03	0.04	0.05	0.06	0.07	0.08	…
*x*	12	12.867	13.278	13.26	12.854	12.119	11.118	9.9238	8.6025	…
*y*	23	19.222	15.176	11.056	7.0527	3.3352	0.036116	-2.768	-5.0471	…
*z*	56	57.125	57.847	58.047	57.688	56.806	55.503	53.898	52.114	…

### 2.2 Test of a sudden change of the Lorenz system

**Fig 1:**
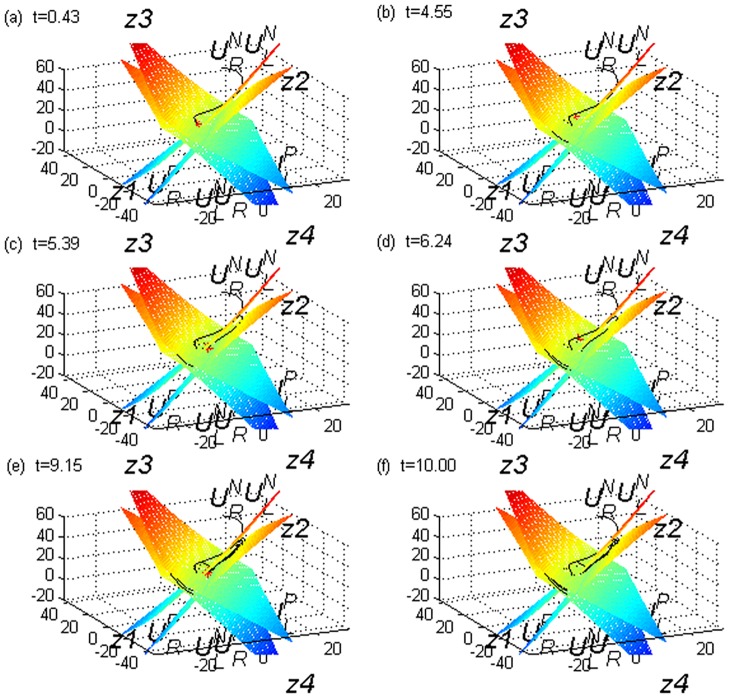
Path of Lorenz system. (a). t = 0.43. (b).t = 4.55.(c).t = 5.39.(d).t = 6.24.(e).t = 9.15.(f).t = 10.00.

As a test, let the initial field of Eq (3) be(12, 23, 56), the integral step 0.01, and the integral interval [0,10], according to the method in[16]. By viewing the path of the Lorenz system, we can observe that there are five sudden changes. In detail, from the initial field point, the path runs through the boundary surface and into the left equilibrium point region. This is the first sudden change, which occurs at approximately 0.43; see Fig 1(a). This sudden change is the basic feature of a dynamical system. All paths eventually converge to the attractor of the Lorenz system, which is the left or right equilibrium point region, informally referred to as beautiful butterfly wings. This is the global stability of a dynamical system. The path revolves around the left equilibrium point for six cycles, then it runs through the boundary surface and enters the right equilibrium point region. This is the second sudden change, which occurs at approximately 4.55; see Fig 1(b). The path revolves around the right equilibrium point for one cycle, runs through the boundary surface, and enters the left equilibrium point region. This is the third sudden change, which occurs at approximately 5.39; see Fig 1(c). The path revolves around the left equilibrium point for one cycle, runs through the boundary surface, and enters the right equilibrium point region. This is the fourth sudden change, which occurs at approximately 6.24; see Fig 1(d). The path revolves around the right equilibrium point for four cycles, runs through the boundary surface, and enters the left equilibrium point region. This is the fifth sudden change, which occurs at approximately 9.15; see Fig 1(e). Fig 1(f) is the image at 10.0. In this test, there are five sudden changes, which consist of four real sudden changes andone false sudden change(i.e.,*t* = 0.43; this is not what we want to detect). We propose a new methodtodetect these four sudden changes, which is more convenient and quicker, has better operability and maneuverability, and can better reflect the path’s overall nature when compared with previous methods. Additionally, it can be applied to some high-dimensional field time series to detect sudden changes, such as a temperature field and global atmospheric pressure field.

### 2.3 From a vector time seriesto a time series

For a vector, both the direction and length are considered. We do the inner product of the vector time series Eq (4) and reference vector *β* = (*k*, *l*, *m*), and construct the time series, as follows:

$$d_{i}^{\left(k,l,m\right)}=\alpha_{i}\cdot \beta \equiv kx_{i}+ly_{i}+mz_{i},$$ (5)

**Fig 2:**
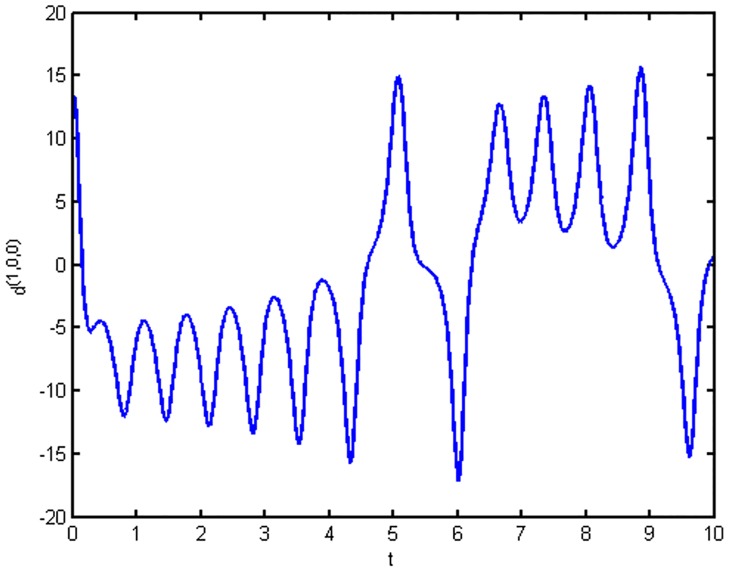
Time series {*d*^(1,0,0)^_*i*_} of reference vector *β* = (1,0,0).

As the geometric meaning of the inner product is the projection, this is equivalent to projecting vector *α*_*i*_ = (*x*_*i*_, *y*_*i*_, *z*_*i*_) onto reference vector *β* = (*k*, *l*, *m*). It is clear that both the direction and length of the vector are taken into account. Where the superscript (*k*, *l*, *m*) indicates that the time series is constructed using reference vector *β* = (*k*, *l*, *m*) and *i* denotes the time point of the time series; mathematically, it is the sequence item number. Thus, we obtain the time series. Consider reference vector *β* = (1,0,0). The angle between the path and vector *β* = (1,0,0) is large; the larger the angle, the better the detection. We perform the inner product and obtain a time series denoted as {*d*^(1,0,0)^_*i*_}, shown in Fig 2:

$$d^{\left(1,0,0\right)}{}_{0}=x_{0},\;d^{\left(1,0,0\right)}{}_{1}=x_{1},\;d^{\left(1,0,0\right)}{}_{2}=x_{2},\;\ldots ,\;d^{\left(1,0,0\right)}{}_{n}=x_{n},\;\ldots$$ (6)

If we take different reference vector *β* = (*k*, *l*, *m*), this shows projecting the vector time series Eq (4) on different vector, and can construct different time series Eq (5), which will result in different conclusions about the sudden change of the vector time series; thus, the selection of reference vector *β* = (*k*, *l*, *m*) is important.

**Fig 3:**
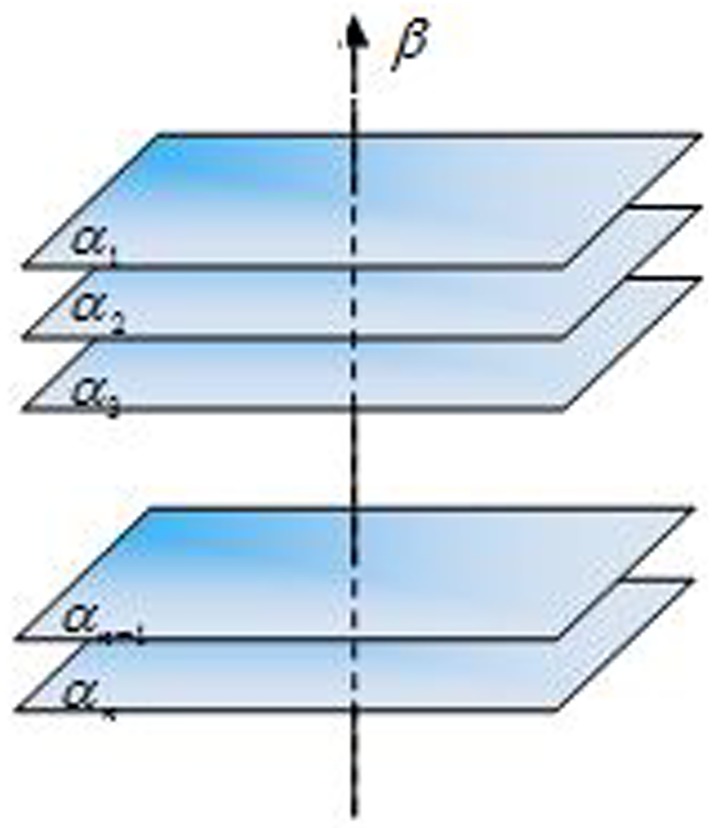
Concept map of the detection method of a sudden change of the vector time series.

To understand the method more clearly, consider the conceptmapin Fig 3, where the surfaces *α*_1_, *α*_2_, *α*_3_, …, *α*_*n*_ are vector time series. Vector *α*_*i*_ is projected onto reference vector *β*. The nature of the vector time series is mainly preserved, but the vector time series is simplified.

### 2.4 Detection method of a sudden change of the vector time series

We use the sliding *t*-test methodto detect the sudden change of Eq (6). The sequences before and after the reference point are *a*_1_ and *a*_2_, their lengths are *n*_1_ and *n*_2_, respectively, their mean values are $\bar{a_{1}}$ and $\bar{a_{2}}$, respectively, and their variances are $s_{1}^{2}$ and $s_{2}^{2}$, respectively, such that[31]

$$t=\frac{\bar{a_{1}}-\bar{a_{2}}}{s\cdot \sqrt{\frac{1}{n_{1}}+\frac{1}{n_{2}}}}$$ (7)

$$s=\sqrt{\frac{n_{1}s_{1}^{2}+n_{2}s_{2}^{2}}{n_{1}+n_{2}-2}}.$$ (8)

**Fig 4:**
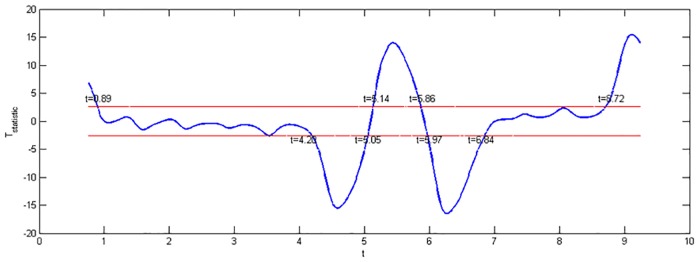
Statistics *T*_statistics_.

The degree of freedom required to construct *T*_statistics_ is 150. The blue line in Fig 4 is sequence *T*_statistics_, the red line is *t* = ±2.609, which exceeds the 0.01 significance level, and the domains beyond the red line are sudden change intervals. The start and end times are marked at the corresponding positions. We can observe from Fig 4 that the blueline and red line intersect for the first time at t = 0.89, which corresponds to t = 0.43. This is when the Lorenz path enters the attractor. The blue line and red line have only one intersection point, but this is not what we want to detect. The time intervals when the blue line and red line intersect for the second, third, fourth, and fifth times are[4.20,5.05], [5.14,5.86], [5.97,6.84], and [8.72,9.25], respectively, as shown in Fig 4, and the sudden changes are at t = 4.55, 5.39, 6.24, and 9.15, respectively, as shown in Fig 1; these times clearly belong to the aforementioned intervals. These results show that our method can detect all the sudden change times of Eq (4). The method is effective for Eq (4), and is feasible and operational. Table 2 shows the sudden change times from Section 2.2 and a comparison.

**Table 2:** Time intervals detected according to reference vector *β* = (1,0,0).

The sudden change time	0.43	4.55	5.39	6.24	9.15
The time interval detected	[0.76,0.89]	[4.20,5.05]	[5.14,5.86]	[5.97,6.84]	[8.72,9.25]
Result	------	Fine	Fine	Fine	Fine

### 2.5 Summary of the detection method

For a general field time series Eq (1), mathematically, it is also a vector time series. Thus, the method applied to the vector time series canbe applied to the field time series. Our proposed method can be used to detect a sudden change of a field time series.

The specific method is as follows: First, select an appropriate reference vector, such as vector *β*. The selection of a reference vector does not only involve considering the geometric and topological characteristics of the time series, but also considering the physical properties usedto generate the time series. Second, perform an inner product to construct a time series based on the reference vector. Finally, use the sliding *t*-test method or another method to detect a sudden change of the time series. A sudden change of the time series is also a sudden change of the field time series.

## 3 Experiment for a sudden change of a meteorological field

We used the proposed method to detect a sudden change of a meteorological field time series, which is the original space-time field time series. We constructed the time series and detected a sudden change. Methods used to study the meteorological field are typically various indices that reduce the original space-time field dimensions.

We compared the inner-product time seriesobtainedby the new method with the corresponding meteorological index and discuss their correlations. We detected the sudden change of these two time series using the sliding *t*-test method and checkedour method’s effectiveness, practicability, and universal adaptability. We selected the sea level pressure(SLP) field and sea surface temperature field of the National Oceanic and Atmospheric Administration (NOAA). The former corresponds to the Aleutian low index and the latter corresponds to the PDO index. The time interval is 1948–2015for each year and January 1854–May 2016 for each month.

### 3.1 Aleutian low index

**Fig 5:**
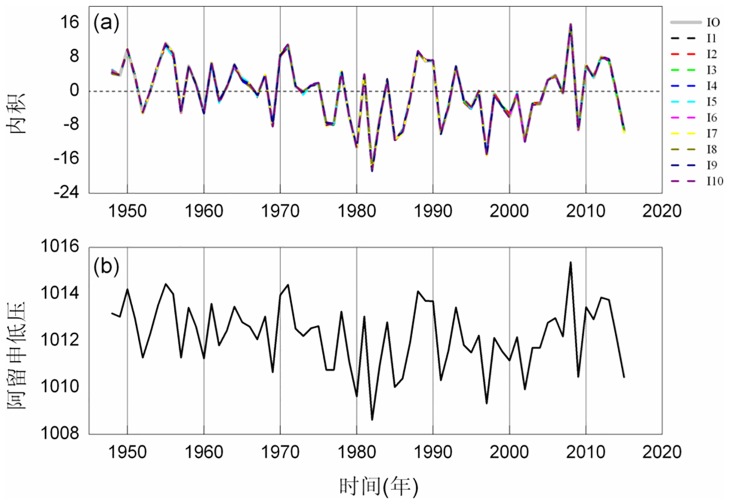
Innerproduct time series and the Aleutian low time series: (a) the inner-product time series (b) the Aleutian low time series.

The Aleutian low appears in the northern hemisphere in winter. It is also semi-permanent and a pan-Arctic cyclone whose center is located near to the Aleutian Islands in the northern Pacific Ocean. Its intensity and position can reflect weather and climate conditions in the northern hemisphere. We used the Aleutian low index proposed by Trenberth in 1990: the average value of the SLP in the north Pacific region (147.5E~122.5W, 27.5N~72.5N) [32]. The inner product time series was structured on the space-time field in the region. The reference vector was produced by a random function using the change of amplitude. We made the magnitude of the variance of the reference vector consistent with the magnitude of the variance of the original field of the SLP. The gray line in Fig 5(a) shows the inner product time series. The other colored lines were obtained from different random reference vectors. We know that although the reference vectors are different, the inner product time series are consistent; the error is approximately 0.1. We compared the inner product time series and the Aleutian low time series and observed that they are almost consistent with the time evolution and have a similar evolutionary structure. The correlation coefficient between them is 0.999; see Fig 5. The two methods used for construction are completely different. From the construction method, we can observe that the former uses the projection, it is a geometric topological method, and the latter calculates the mean value, it is a statistical method. However, the results reach the same conclusion independently.

**Fig 6:**
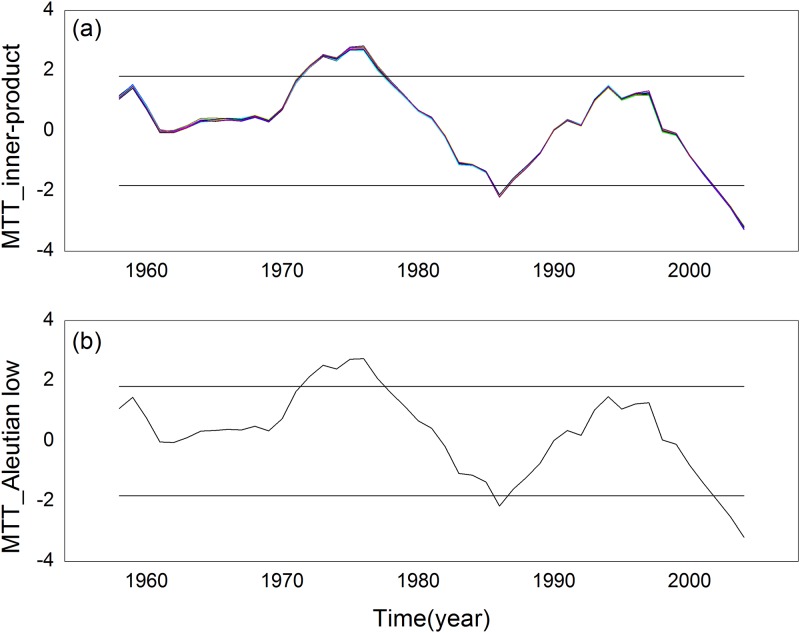
Test results, where the gray line is the 0.05 significance test. (a) the inner-product time series (b) the Aleutian low time series.

We detected the inner product time series and the Aleutian low index structure usingthe sliding *t*-test method. The results are shown in Fig 6. Their sudden changes are in good agreement with each other. A remarkable sudden change occurred in the mid and late 1970s, and we also detected a sudden change of the inner product time series and the Aleutian low time series in the mid and late 1970s. Moreover, the inner product time series has phase inversion. Additionally, the results of the innerproduct time series show that there was asudden change in the mid-1980s. We compared the time series and observed a change from a negative phase to a positive phase briefly in the mid-1990s. The inner product time series has a positive phase to negative phase conversion; however, the results show that this conversion is not significant.

### 3.2 Pacific interdecadal oscillation (PDO) index

**Fig 7:**
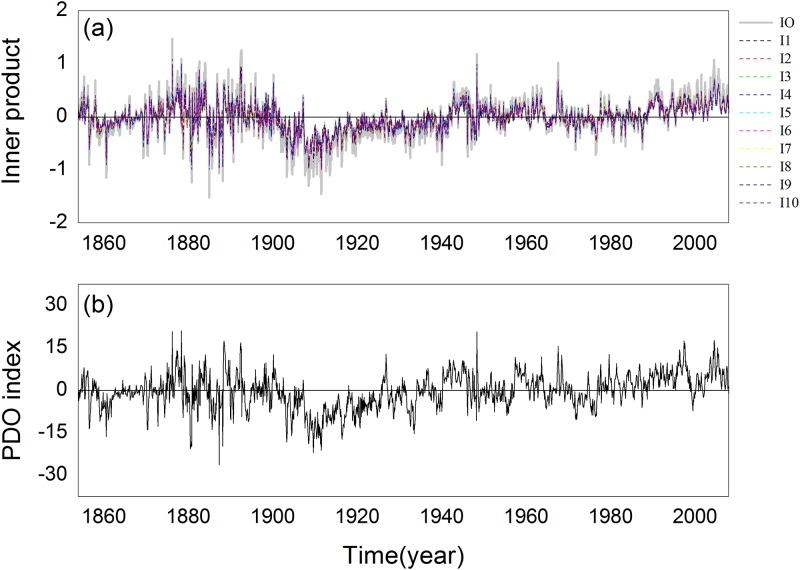
Inner product time series structure and PDO index series: (a) inner product time series (b) PDO index series.

The PDOis an important variable that is a reflex of the decadal signal of the north Pacific [33]. It is the time coefficient of the first mode of the empirical orthogonal function of the sea surface temperature (SST) of the Pacific Ocean north of 20N; the SST is the original field. We use the proposed methodto obtain the innerproduct of the time series. The reference vector is produced by a random function, similar to Section 3.1. The inner product time series structured using different reference vectors and PDO index series are given in Fig 7. We can observe that they have nearly the same evolutionary structure and their correlation coefficient is 0.793. From the construction method, we can observe that the construction mechanisms are not the same. The former is a projection method and the latter uses modal coefficients.

**Fig 8:**
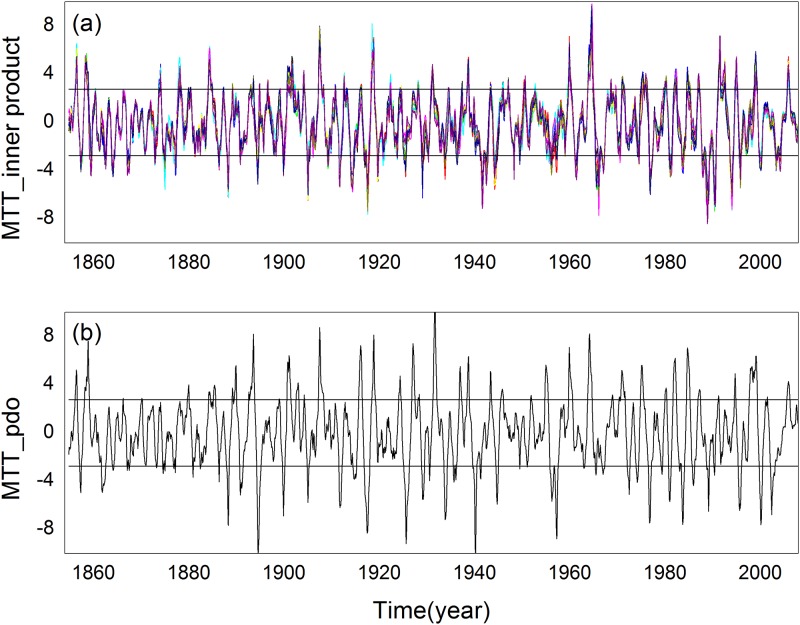
Sudden change detection using the sliding t-test method: (a) sudden change detection of the innerproduct time series(b) sudden change detection of the PDO index series.

The inner product time series and PDO index series perform sudden change detection using the sliding *t*-test method; see Fig 8. We can observe that they have similar sudden change results. Sudden change detection of the inner producttime series exceeded 0.01 degrees, whichis far less than the detection results of the PDO index series. The proposed method is more conducive to distinguishing real sudden change. In approximately 1900 and 1920, sudden changes were identified and the inner product time series and PDO index series transformed from a positive phase to negative phase and negative to positive phase for these years, respectively. In approximately 1930, two series did not undergo a significant transformation. The PDO time series hada false detection, but the inner product time series did not. We investigated the reason and found that the inner product time series was obtained using the reference vector. This partly reduces the probability of the appearance of abnormal values; however, the PDO series has more abnormal values, which leads to more false detection.

### 3.3 Summary

We constructed random reference vectors and performed the inner product with the actual space-time field to obtain the inner product time series. Thus, we transformed the original complex field time series into a time series and obtained the sudden changes of the original space-time field time series using sudden change detection of this time series. By detecting the original meteorological fields of the Aleutian low index and PDO index, and comparing them with their own index time series, we found that the proposed method effectively detected the sudden change of the field time series, and to a certain extent, also eliminated false detection, which is caused by the appearance of abnormal values. Simultaneously, the method can be applied to any field time series and has good general applicability; for a given field time series, can we detect its sudden changes.

## 4 Conclusions

Using the numerical solution of the Lorenz system, the surfacedemarcating the regions to the left and right of the equilibrium point of the Lorenz system, and some mathematical techniques, we proposed a new method to detect a sudden change of the field time series. The method has good practicability and universality, but requires certain mathematical and physical techniques that are based on an adequate understanding the geometric characteristics and physical properties of the field time series. Whether using the test of the Lorenz system or the test of the practical meteorological field time series, a sudden change can be detected. Thus, our proposal is innovative, both in the method and theory.

Using this method to detectthe meteorological field time series, a reference vector was chosen randomly. We can be certain that if the reference vector is selected properly, we can obtain reliable sudden change detection results, and this requires a more accurate understanding of the physical process of the atmosphere. Additionally, it requires anunderstandingof the geometric topological features of atmospheric motion equations.

The sudden change is the result of the mathematics and physics fundamental research, but it can knowand predict the development of complex systems, for instance, the economic performance, the biological change (especially genetic mutations), the earthquake early warning[34–40]. The method and conclusion in this paper may improve the prediction of these complex systemsin big data.

## Supporting information

S1 Dataset slp.rar–the slp.zip is the Aleutian low data. (RAR)

S2 Dataset sst.rar—the sst.rar is the PDO data. (RAR)
